# Mutations of the MAPK/TSC/mTOR pathway characterize periventricular glioblastoma with epithelioid SEGA-like morphology–morphological and therapeutic implications

**DOI:** 10.18632/oncotarget.27005

**Published:** 2019-06-18

**Authors:** Maria-Magdalena Georgescu, Yan Li, Mohammad Zahidul Islam, Christina Notarianni, Hai Sun, Adriana Olar, Gregory N. Fuller

**Affiliations:** ^1^ Department of Pathology and Pathobiology and Feist-Weiller Cancer Center, Louisiana State University, Shreveport, LA 71103, USA; ^2^ Department of Neurosurgery, Louisiana State University, Shreveport, LA 71103, USA; ^3^ Department of Pathology and Laboratory Medicine and Neurosurgery, Medical University of South Carolina and Hollings Cancer Center, Charleston, SC 29425, USA; ^4^ Department of Pathology, The University of Texas MD Anderson Cancer Center, TX 77030, USA

**Keywords:** epithelioid glioblastoma, SEGA-like, mTOR, MAPK, 4E-BP1

## Abstract

Epithelioid glioblastoma is a recognized glioblastoma variant, recently added to the World Health Organization brain tumor classification, with similar prognosis as the classic variant and B-Raf V600E mutations in 50% of the cases. We identified a new subset of epithelioid glioblastoma with periventricular location and subependymal giant cell astrocytoma (SEGA)-like morphology. Genomic profiling of these tumors revealed driver mutations in *NF1*, subclonal mutations in *TSC1*, and a novel driver mutation in *MTOR*, suggesting upregulation of the MAPK/TSC1/mTOR pathway. Strong mTOR activation was confirmed by immunohistochemistry for the mTOR kinase target 4E-BP1. *TSC1* and *MTOR* mutations have been previously described in low-grade glioma, such as SEGA, and focal cortical dysplasia, respectively, that display large cells with abundant cytoplasm, most likely resulting from the biogenetic signaling of mTOR. Unlike these, the mutations in SEGA-like glioblastoma occurred in the context of other genetic aberrations present in high-grade neoplasms, including in the *CDKN2A/B*, *PIK3R1*, *PIK3CA* and *EGFR* genes. For one patient with two temporally distinct specimens, the subclonal *TSC1* pathogenic mutation was detected only in the specimen showing SEGA-like morphology, indicating requirement for mTOR activation as trigger for specific epithelioid/SEGA-like morphology. As FDA-approved kinase inhibitors are available and target many steps of the MAPK/mTOR pathway, recognition of this new subset of periventricular high-grade gliomas with clear phenotypic-genotypic correlates is essential for prompt biomarker testing and appropriate targeted therapeutic management of these patients.

## INTRODUCTION

Glioblastoma is the most frequent and aggressive glial tumor in adults, with an incidence of 3–4 cases per 100 000 population, and a median survival of 1.3 years [1]. Because of its dismal prognosis, an intense search for new therapies has been linked to an effort to discover new therapeutic targets. An individualized approach has been increasingly adopted in oncology, recognizing the diversity of tumors previously diagnosed under the same morphologic umbrella. In glioblastoma, stratification criteria by morphological-molecular correlations are now sought after at the time of the initial diagnosis in order to inform timely therapeutic decisions, with critical implications for patient survival. Three morphologic variants of glioblastoma are recognized in the 2016 World Health Organization (WHO) Classification of Tumors of the Central Nervous System (CNS): gliosarcoma, giant cell glioblastoma, and epitheliod glioblastoma [1]. Epithelioid glioblastoma, the latest addition incorporated into the 2016 classification, has been shown to harbor the B-Raf V600E mutation in 50% of the cases [2]. Importantly, the B-Raf V600E mutation constitutively activates the mitogen-activated protein kinase (MAPK) signaling pathway in a large variety of cancers and is a targetable mutation for which FDA-approved drugs are already available [3]. In gliomas, B-Raf V600E is also well represented in predominantly cortical, young-onset, low-grade, morphologically-similar entities, such as ganglioglioma, pleomorphic xanthoastrocytoma and astroblastoma [4, 5]. These may be occasional components of epithelioid glioblastoma in some instances of anaplastic transformation of an initial low-grade neoplasm [2, 6]. Interestingly, another low-grade glioma (WHO grade I) with epithelioid morphology is subependymal giant cell astrocytoma (SEGA). SEGA almost always occurs in tuberous sclerosis patients with a germline mutation in the tuberous sclerosis complex (TSC) genes, and, as its name implies, it is always periventricular [1, 7].

The MAPK pathway is a linear pathway that starts downstream from receptor tyrosine kinases (RTKs), such as epidermal growth factor receptor (EGFR), with the activation of the small GTP-ase protein Ras that, in turn, binds and activates the Raf family of serine-threonine protein kinases, including B-Raf [8]. The Raf kinases are the upmost in a series of three consecutive levels of protein kinases that are activated by the upstream level kinase through protein phosphorylation and subsequent conformational change, with the second level being the MEK kinases, and the third, the ERK kinases, which are also called MAPKs (see also Figure 5). ERK1/2, as well as their direct target p90 ribosomal S6 kinase 1 (RSK1), phosphorylate TSC2, leading to the inactivation of TSC [9]. The TSC heteromeric complex formed by TSC1, TSC2 and TBC1D7 acts as a GTPase activating protein (GAP) for the small Ras-related GTPase Rheb, which, in active GTP-bound form, binds and activates mTOR. Through many converging modulatory loops, TSC represents the main upstream negative regulator of mTOR. As terminus for many pathways, mTOR serves as primary regulator of cell growth in response to cellular nutrition, energy levels and growth factor stimulation [7].

In this study, we delineated a new subset of periventricular glioblastoma that exhibit a plump SEGA-like/epithelioid morphological phenotype and bears mutations that result in mTOR activation, including mutations of *TSC1* and *MTOR* itself. These genetic alterations have been previously seen only in low-grade CNS lesions, such as SEGA and cortical dysplasia [7, 10]. Since therapeutic inhibitors are already available, we propose testing for these markers and therapeutic targets in all periventricular epithelioid high-grade gliomas.

## RESULTS

### Clinical-pathologic correlations in three patients with periventricular epithelioid/SEGA-like high-grade glioma

Three patients with IDH and histone H3 wild-type high-grade diffuse glioma with epithelioid morphology were analyzed in this study and their demographic data are presented in Table 1.

**Table 1:** Patient clinical data

Case	Sex/age years	Location	Size cm	Diagnosis WHO grade	Surgery	Therapy	Rx Rec months	Survival months
#1	M60	R thalamus	5 × 3	AA III	Subtotal	XRT	7	12
#2	F65	L peri-LV	7 × 3	GBM IV	Subtotal	None	0	3
#3	M46	R LV atrium	2.5	GBM IV	Total	XRT, TMZ, GK	11, 8	28^**1**^

Abbreviations: M, male; F, female; R, right; L, left; LV, lateral ventricle; AA, anaplastic astrocytoma; GBM, glioblastoma; XRT, radiotherapy; TMZ, temozolomide; GK, gamma knife; Rx Rec, radiologic recurrence.

^1^The patient was transferred to hospice.

**Figure 1:**
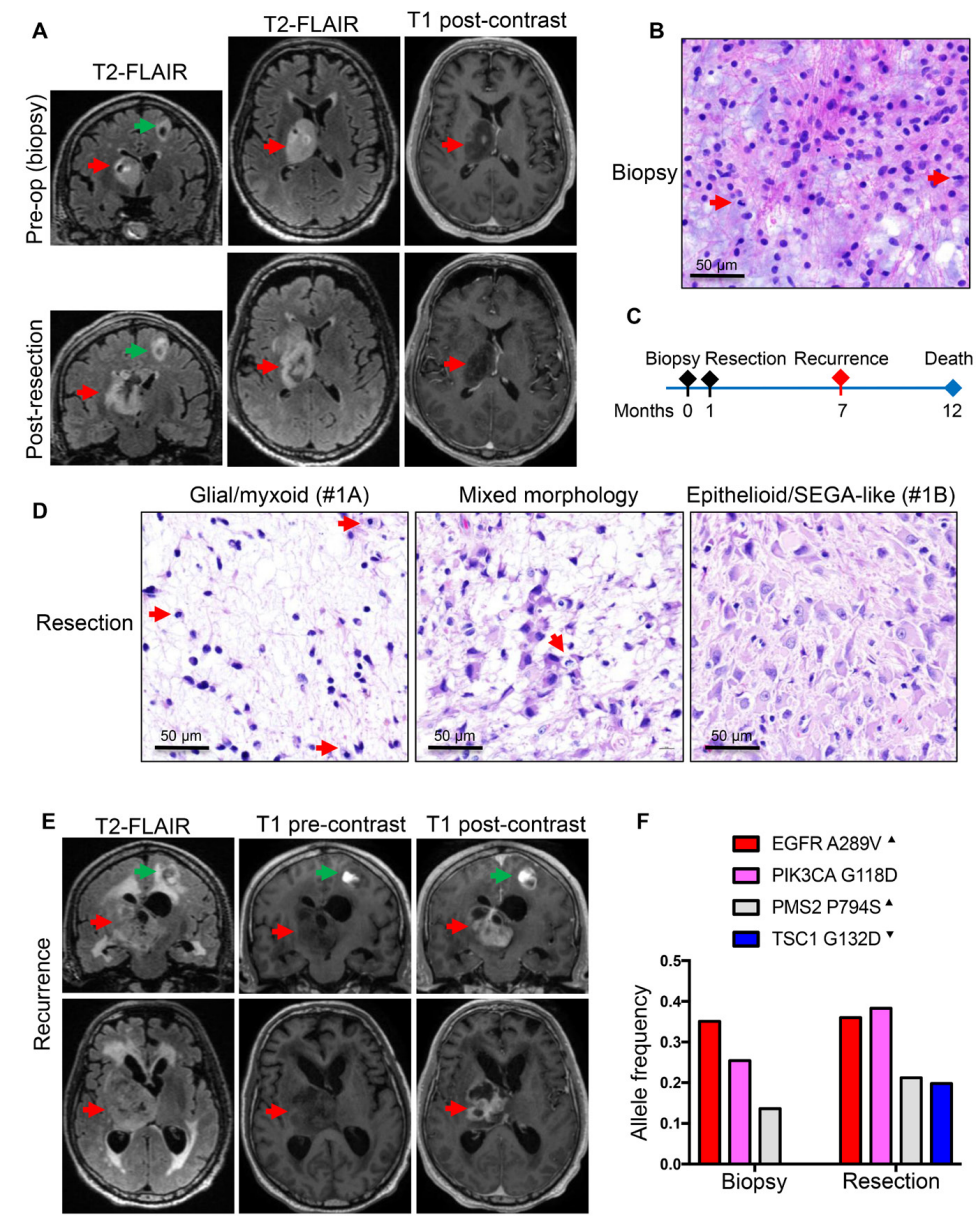
Patient 1: Aggressive thalamic anaplastic astrocytoma with *TSC1* mutation and 1-year survival. (**A**) Pre-biopsy and post-resection MRI coronal and axial T2W-FLAIR and axial T1W post-contrast images showing a large right thalamic mass (red arrows) with hyperintensity on FLAIR images and only punctate enhancement on post-contrast images. A second smaller cystic mass (green arrows) is apparent in the left frontal area. (**B**) Biopsy H&E shows neoplastic astrocytes with large, round nuclei, some undergoing mitotic division (red arrows), within a myxoid background. (**C**) Time course of the patient’s clinical progression. (**D**) H&E of CUSA specimen shows areas similar to the biopsy (left panel), composite areas containing both morphologic patterns (middle panel), and extensive areas of SEGA-like morphology (right panel). Mitotic figures are indicated by red arrows. (**E**) Six-months post-resection coronal and axial T2W-FLAIR and pre- and post-contrast T1W sequences show large rim-enhancing recurrence. (**F**) NGS of the biopsy and resection reveals *EGFR* and *PIK3CA* driver, and *PMS2* subclonal, mutations in the tumor regardless of morphology, and *TSC1* mutation confined to the tumor with SEGA-like component. Superscript arrowhead indicates gain or loss of the chromosomal locus for the respective gene (see also Supplementary Table 2).

The first patient, a 60-year-old white man, without significant past medical history, presented with memory issues and fatigue for a few months prior to a fall from a ladder in which he fractured his right femur and acetabulum. Computed tomography (CT) imaging showed a 5 × 3 cm right thalamic mass with focal hemorrhage and calcification. Brain magnetic resonance imaging (MRI) showed strong hyperintensity on T2-weighted FLAIR images, but only limited punctate enhancement on post-contrast T1-weighted images (Figure 1A, upper panels). A second smaller cystic mass, measuring 0.9 × 0.8 cm, was detected in the left frontal lobe; this mass was not biopsied. The patient underwent an initial biopsy of the thalamic mass, for which the diagnosis was anaplastic astrocytoma, IDH and histone H3 wild-type, WHO grade III (Figure 1B). The Ki-67 proliferation index was 12% (Table 2). A subtotal resection followed shortly (Figure 1C), encompassing the areas of punctate enhancement (Figure 1A lower panels). Interestingly, the pathology showed two histologic patterns: a first pattern of neoplastic astrocytes with large, round nuclei embedded in an abundant myxoid extracellular matrix (Figure 1D, left panel; Supplementary Figure 1, SEGA-like case 1A), and a second pattern of a solid growth composed of larger and polygonal SEGA-like astrocytes with abundant cytoplasm (Figure 1D, right panel; Supplementary Figure 1, SEGA-like case 1B). Mitotic figures were scattered, with up to 3 per high-power field, but no necrosis or microvascular proliferation were seen in the relatively small specimen. The Ki-67 proliferation index was up to 20% (Table 2). Of note is that the fragment of tumor obtained by biopsy contained only areas of the first pattern, without SEGA-like morphology (Figure 1B). The tumor pursued an aggressive course, inducing paralysis and rapid transition from punctate to ring enhancement on MRI (Figure 1E). The patient died a few months later, 1 year after initial diagnosis.

**Table 2:** Histology-molecular pathology correlates

Case/Specimen	Morphology	Necrosis Thrombi	Imm infiltr	Ki-67 index	Chr loss^1^	Mutations^2^ MAPK/mTOR	Mutations^2^ Other
#1/Biopsy	Glial/ myxoid	-	-	12%	NP	-	EGFR A289V PIK3CA G118D
#1/Res	Epithelioid/ SEGA-like	-	-	20%	del9 del14q del22	TSC1 G132D	EGFR A289V PIK3CA G118D
#2/Res	Gemistocytic / Epithelioid/ SEGA-like	+++	+	13.3%	del9p del14 del22q	NF1 G1912^*^ NF1 I2130/fs	PIK3R1 K459del CDKN2A/B loss
#3/Res	Epithelioid/ SEGA-like	++	+++	18.5%	del9 del14 del22	MTOR Q2499R TSC1 R786^*^	RB1 R255^*^ gain7/del10

Abbreviations: Res, resection; Imm Inflitr, immune infiltration with small lymphocytes; Chr, chromosome; NP, not performed.

^1^Only common CN alterations between the cases are shown here. The complete list of CN alterations is shown in Supplementary Table 2.

^2^Clonal mutations are underlined, and subclonal mutations are in normal script. The complete description of the mutations is rendered in Supplementary Table 3.

**Figure 2:**
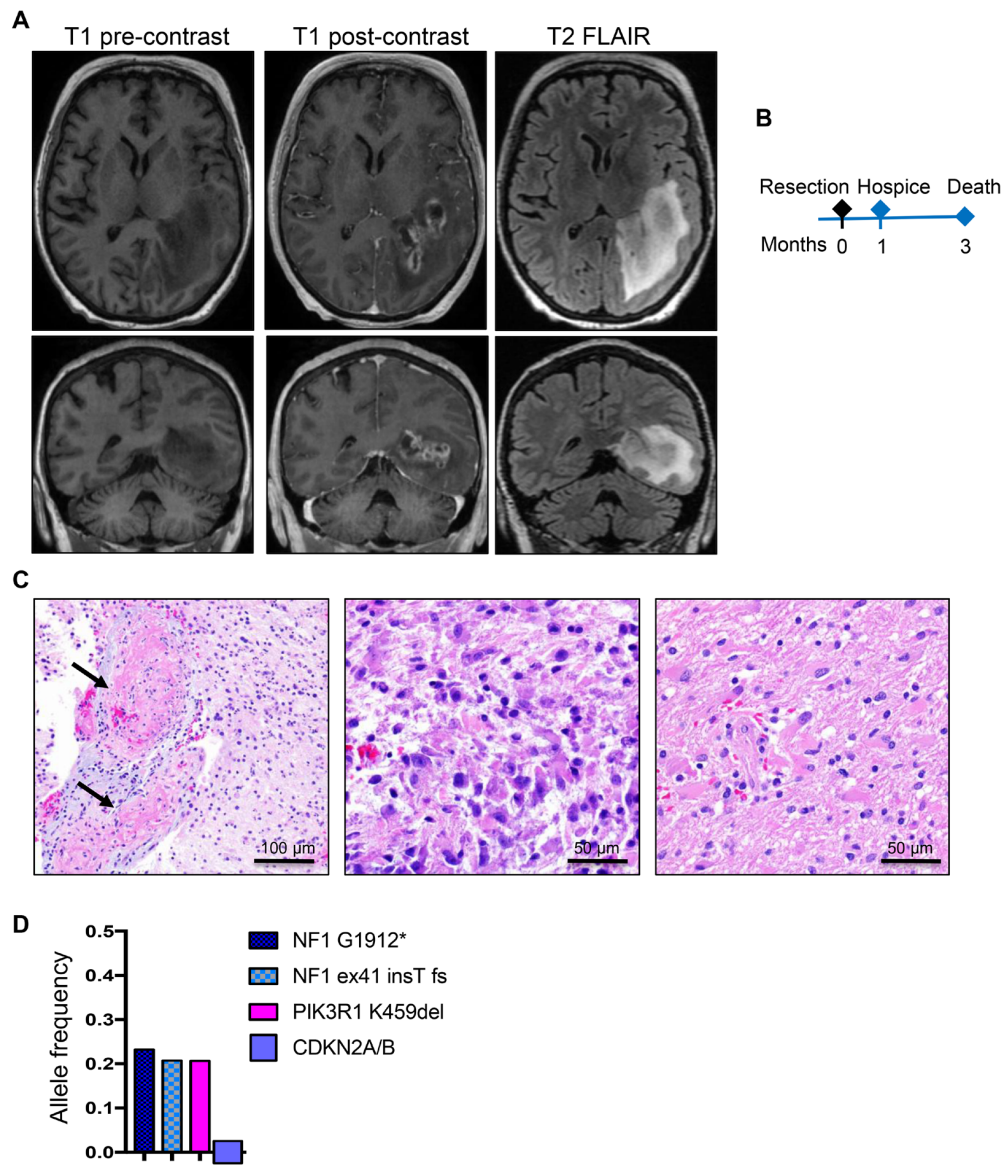
Patient 2: Aggressive periventricular glioblastoma with *NF1* mutations and 3-month survival. (**A**) Preoperative axial and coronal T1W pre-contrast and post-contrast and T2W-FLAIR MRI images showing a large, left periventricular, ring-enhancing mass with surrounding infiltration and edema on FLAIR images. (**B**) Time course of clinical progression. (**C**) Resection H&E shows massive vessel thrombosis (arrows). Epithelioid/gemistocytic neoplastic cells are clustered in small foci (middle panel) and diffusely invading the neuropil (right panel). (**D**) NGS reveals a *PIK3R1* and two truncating *NF1* driver mutations, and SNP-microarray shows homozygous *CDKN2A/B* loss.

The second patient, a 65-year-old white woman, had a past medical history of non-malignant breast tumor, status-post right mastectomy without chemo- or radio-therapy, 3 years prior to developing expressive aphasia and unrelenting headache for 2 weeks followed by nausea and vomiting. On ophthalmic examination, the patient had right homonymous hemianopsia. MRI showed a large (7 × 3 cm), elongated, rim-enhancing mass located in the left temporo-occipital region, between the atrium and the temporal horn of the lateral ventricle, infiltrating the ependyma of the atrium and the hippocampus, and surrounded by extensive vasogenic edema (Figure 2A). Subtotal resection was performed (Figure 2B and Table 1), and histopathologic examination revealed a lesion with extensive ischemic necrosis and many vessels obstructed by fibrin microthrombi (Figure 2C, left panel). Epithelioid/gemistocytic tumor cells were seen either in clustered foci or, more often, infiltrating the parenchyma (Figure 2C, central and right panels, respectively; Supplementary Figure 1, SEGA-like case 2). Scattered mitotic figures were present but they were not numerous. The maximum Ki-67 proliferation index was 13.3% (Table 2). Immunohistochemistry (IHC) showed positive GFAP expression, but no IDH1-R132H, histone H3 K27M or p53 staining of neoplastic cells, and the tumor was diagnosed as glioblastoma, WHO grade IV. The patient was placed in hospice one month after the resection and succumbed three months after initial diagnosis (Table 1 and Figure 2B).

**Figure 3:**
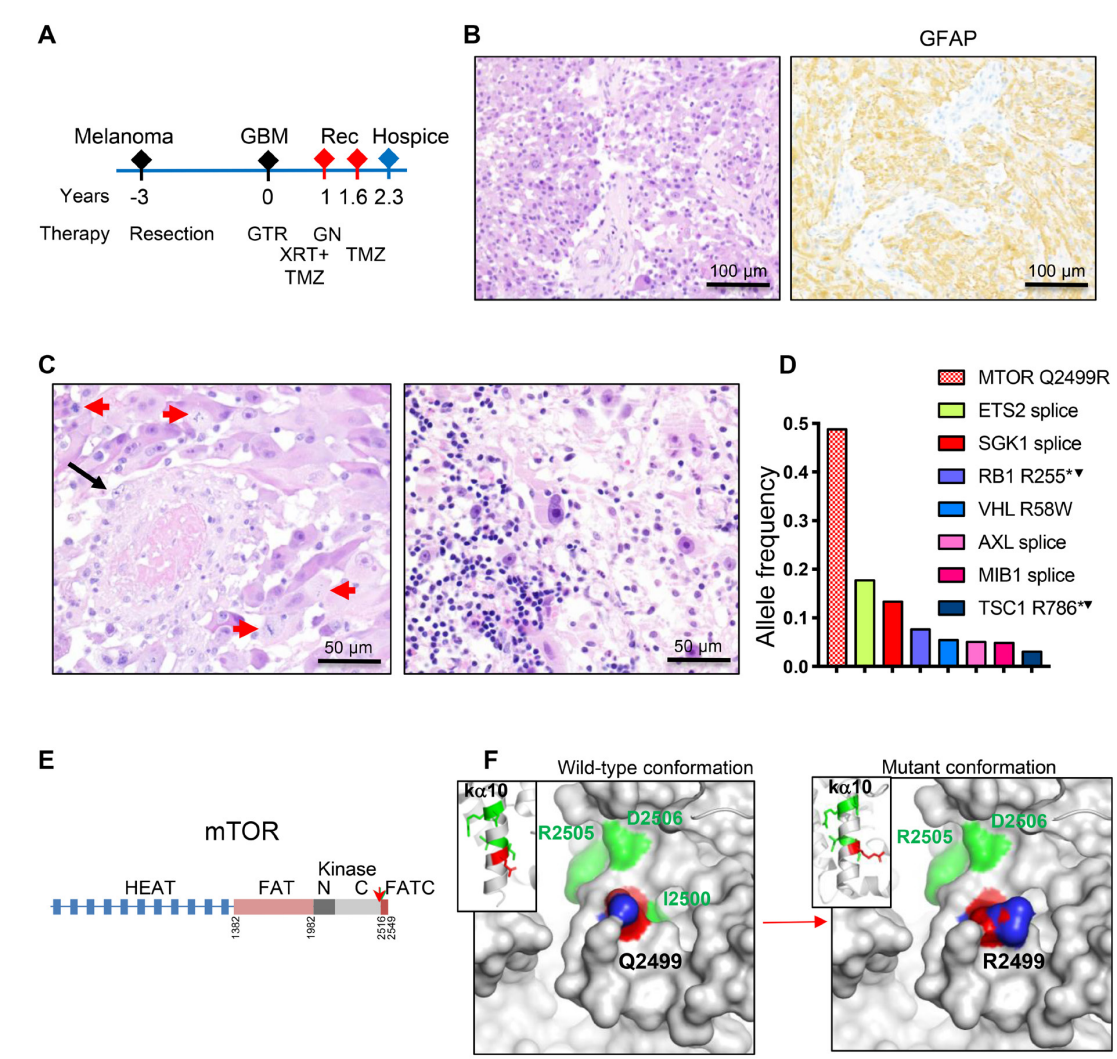
Patient 3: Intraventricular glioblastoma with *MTOR* mutation and over 2-year survival. (**A**) Time course of clinical progression, including the prior resection for thigh melanoma. Abbreviations: Rec, recurrence; GTR, gross total resection; GN, gamma knife; XRT, radiotherapy; TMZ, temozolomide. (**B**) Resection H&E and IHC show diffuse SEGA-like morphology of neoplastic cells that are positive for GFAP. (**C**) H&E also shows vessel thrombosis (black arrow), numerous mitotic figures (red arrows) and prominent infiltration with lymphocytes (right panel). (**D**) NGS reveals a missense *MTOR* driver mutation, and multiple subclonal mutation. Superscript arrowhead indicates loss of the chromosomal locus for the respective gene (see also Supplementary Table 2). (**E**) Structural mapping of the novel mTOR Q2499R mutation. Domain map of mTOR: HEAT (Huntigtin, elongation factor 3, protein phosphatase 2A, TOR1) repeats, FAT (FRAP, ATM, TRRAP) domain, N- and C-terminal lobes of the kinase domain and FATC C-terminal domain. Amino acid boundaries are indicated. The Q2499R mutation is shown by red arrow. (**F**) Ribbon (insets) and surface 3D representations of mTOR last alpha helix of the kinase domain (ka10) oriented to view the positioning of 5 backbone residues mutated in human cancer: Q2499 in red, and I2500, I2501, R2505 and D2506 in green, as well as and their projection to the surface. The surface representation panels show that the Q2499R mutation induces a significant surface conformational and net charge change, with replacement of the hydrophobic wild-type relief formed by Q2499 and I2500 side chains with a positively-charged patch from the R2499 side chain. The side chain labeling: O atoms in red, N atoms in blue.

The third patient, a 46-year-old white man, had a history of malignant melanoma of the right thigh resected 3 years prior to developing brain symptomatology (Figure 3A). The family history was significant for brain tumor in the patient’s father. MRI showed a 2.5 cm diameter round, contrast-enhancing mass in the right lateral ventricle atrium; gross total resection was subsequently performed (Table 1). Histopathological examination showed a neoplasm with solid architecture composed of SEGA-like epithelioid cells with nuclei displaying large prominent nucleoli (Figure 3B–3C; Supplementary Figure 1, SEGA-like case 3). IHC showed positive labeling of neoplastic cells with GFAP (Figure 3B), with no expression of HMB45, MART1, S100 protein, pancytokeratin, EMA, synaptophysin, IDH1-R132H or histone H3 K27M. The tumor exhibited necrosis and vascular proliferation, and, as in the previous case, vessel thrombosis (Figure 3C, left panel). Mitotic figures were numerous and the maximum Ki-67 proliferation index was 18.5% (Table 1). In contrast to the other two cases, the tumor displayed prominent lymphocytic infiltration (Figure 3C, right panel). The diagnosis rendered was glioblastoma, WHO grade IV. The patient underwent concurrent treatment with radiation and temozolomide, but 11 months later developed radiological recurrence that was treated with gamma knife radiotherapy, and 8 months after that, new radiologic recurrence was seen (Figure 3A and Table 1). He continued treatment with temozolomide. Two years and four months after brain tumor resection, the patient was placed in hospice due to disease progression, and was subsequently lost to follow-up (Table 1).

The morphologic resemblance of these three SEGA-like high-grade gliomas to SEGAs is shown in Supplementary Figure 1, where 4 cases of SEGA are also illustrated. SEGA cells have been shown to express TTF-1, suggesting lineage-restricted histogenesis [11]. In our hands, IHC with a TTF-1-specific antibody has shown moderate staining of subsets of neoplastic cells in SEGA cases, confirming the reported data [11]. In contrast, all three glioblastoma SEGA-like cases were negative for TTF-1 expression (Supplementary Figure 2).

### Molecular pathologic correlations: mutations in *TSC1*, *NF1* and *MTOR* underlie epithelioid/SEGA-like morphology in periventricular glioblastoma

Next generation sequencing (NGS) of the four samples available from the three patients revealed two sets of mutations (Table 2). A first set of mutations mapped to genes encoding proteins activating the MAPK/TSC/mTOR pathway. A second set mapped to genes involved in general tumor pathogenesis. The mutations from the first set were in *TSC1*, *NF1* and *MTOR* (Table 2 and Figures 1F, 2D and 3D).

A *TSC1* mutation in the 1st patient was not detected in the initial biopsy specimen and was present only in the resection specimen at subclonal allele frequency (Table 2 and Figure 1F). The presence of this mutation at subclonal allele frequency correlated with the patchy epithelioid/SEGA-like morphology in the resection specimen (Table 2 and Figure 1D). The G132D missense mutation in the amino (N)-terminus of TSC1 was initially found in patients with TSC syndrome and shown to destabilize TSC1 and prevent the inhibition of mTORC1, leading to mTOR activation [12]. The crystal structure of TSC1 showed that all pathogenic mutations in the N-terminus, including G132D, disrupt the folding of the N-terminal 265-residue globular domain [13]. The tumor also contained two oncogenic missense mutations in EGFR A289V [14] and PIK3CA G118D [15]. These were somatic mutations, present in both specimens at homozygous clonal allele frequency, indicating that they are driver mutations present in both morphological subpopulations. A forth subclonal missense mutation in PMS2 P794S, previously reported in Lynch syndrome (ClinVar: 3 entries), was also present in both specimens (Figure 1F). The chromosomal analysis showed high copy gain of chromosome 7p containing the *EGFR* locus (Table 2), but also of 7q and chromosome 5 (Supplementary Table 2). Chromosome 20 and 21 gains were specific to this tumor, but the three chromosomal deletions on chromosome 9, 14 and 22 were common to all three cases (Table 2 and Supplementary Table 2). In particular, loss of chromosome 9 indicated LOH for *TSC1* locus at 9q34 in the subset of neoplastic cells with TSC1 D132D mutation (Supplementary Table 2)*.*

The 2nd patient’s tumor showed two *NF1* somatic mutations predicted to truncate the protein, most likely located on different alleles, indicating loss of heterozygosity (LOH) (Table 2 and Figure 2D). A *PIK3R1* oncogenic mutation resulting in an in-frame deletion of K459 from the iSH2 region of the p85α regulatory subunit of the PI3K [16] was also present at similar heterozygous clonal allele frequency as the two *NF1* mutations, indicating that these are driver mutations. Seven additional single nucleotide polymorphisms (SNPs) determining missense mutations in other genes confined only to this case were detected at roughly 0.5 allele frequency, suggesting germline origin. One of these, ATR S902P, was previously described as a somatic mutation in metastatic cancer [17], and could have contributed to the development of this neoplasm. The chromosomal analysis showed only deletions, either focal/segmental for 1p, 4q, 9p, including homozygous *CDKN2A/B* loss, 12q, 21q, 22q or losses of chromosomes 6, 10, 13, 14, 18p and 18q (Table 2 and Supplementary Table 2).

The 3rd patient’s tumor was doubly sequenced in our laboratory and in Tempus laboratory. A missense mutation in mTOR Q2499R was detected in both instances at 0.49 allele frequency for a high tumor content of 80%, and could represent a germline cancer predisposing variant rather than a somatic mutation in this relatively young patient with history of both cutaneous melanoma and glioblastoma (Figure 3D). The mTOR Q2499R mutation maps to the carboxyl (C)-terminal lobe of the kinase domain, ka10 (Figure 3E), and is adjacent to several mutations previously described to activate mTORC1 in cancer [18, 19]. All these residues form the backbone of the most C-terminal alpha helix of the kinase domain (Figure 3F), and are supposed to restrict access to the catalytic site [20]. The change Q2499R induces a significant protein surface alteration, both in conformation and charge distribution, by rendering a net positive charge to the surface (Figure 3F). The tumor showed a relatively high tumor mutational burden, at 5 mutations/megabase DNA (74th percentile Tempus ranking), but all the other variants were detected at relatively low subclonal allele frequency (Figure 3D). These comprised two truncating pathogenic variants—RB1 R255* and TSC1 R786*, one missense probable damaging variant—VHL tumor suppressor R58W [21], and four splice variants of unknown significance in ETS2 transcription factor, SGK1 serum/glucocorticoid regulated kinase 1, AXL RTK and MIB1 (Figure 3D). Whereas these subclonal mutations may have contributed to tumorigenesis, it is most likely the ubiquitous presence of the mTOR Q2499R mutation that may explain the homogeneity of the histological SEGA-like appearance in this tumor. The chromosomal analysis showed gain of chromosome 7 and loss of chromosome 10, which is one of the most frequently seen alterations seen in glioblastoma, as well as loss of chromosomes 6, 13, 14, 16 and 22 (Table 2 and Supplementary Table 2).

To integrate these results, we compared them to those obtained from the NGS of 50 additional adult IDH wild-type glioblastoma cases. Mutations activating the MAPK/TSC/mTOR pathway were present in 10 cases that did not show a primarily periventricular location, although they frequently involved the posterior half of the corpus callosum (4 cases). These consisted of one gliosarcoma case with NRAS Q61K gain of function mutation, one superficial glioblastoma case with oligodendroglial-like morphology and BRAF D594N loss of function mutation that nevertheless activates ERK/MAPK through activation of cRAF [22], and eight cases with *NF1* pathogenic mutations, two of which also containing an additional *MTOR* splice region variant of unknown significance (A1971V; NM004958) or a *TSC2* in frame deletion/insertion (S1282-G1285 delinsR; NM 000548). The neoplasms with *NF1* pathogenic mutations harbored a range of morphologies, from predominantly spindle cell in 5 cases, to giant cell, gemistocytic or epithelioid in one case, each (Supplementary Figure 3). The latter was a very infiltrative superficial left parietal tumor exhibiting classical non-SEGA-like epithelioid morphology and NF1 C167fs truncating mutation with LOH (Supplementary Figure 3A). It was also the only case with classic epithelioid morphology from the additional 50-case cohort. The *MTOR* and *TSC2* mutations co-occurred with *NF1* pathogenic mutations with LOH in two tumors with parietal location and imparted a plump appearance to the spindle cell morphology of the tumors with obvious areas of classical epithelioid morphology (Supplementary Figure 3B–3C). The tumor with MTOR splice variant had areas of perivascular arrangement of the plump spindle cells, a pattern that may be seen sometimes focally in SEGAs (compare Supplementary Figure 3B with Supplementary Figure 1 SEGA#4).

### Activation of mTOR kinase in periventricular SEGA-like glioblastoma

**Figure 4:**
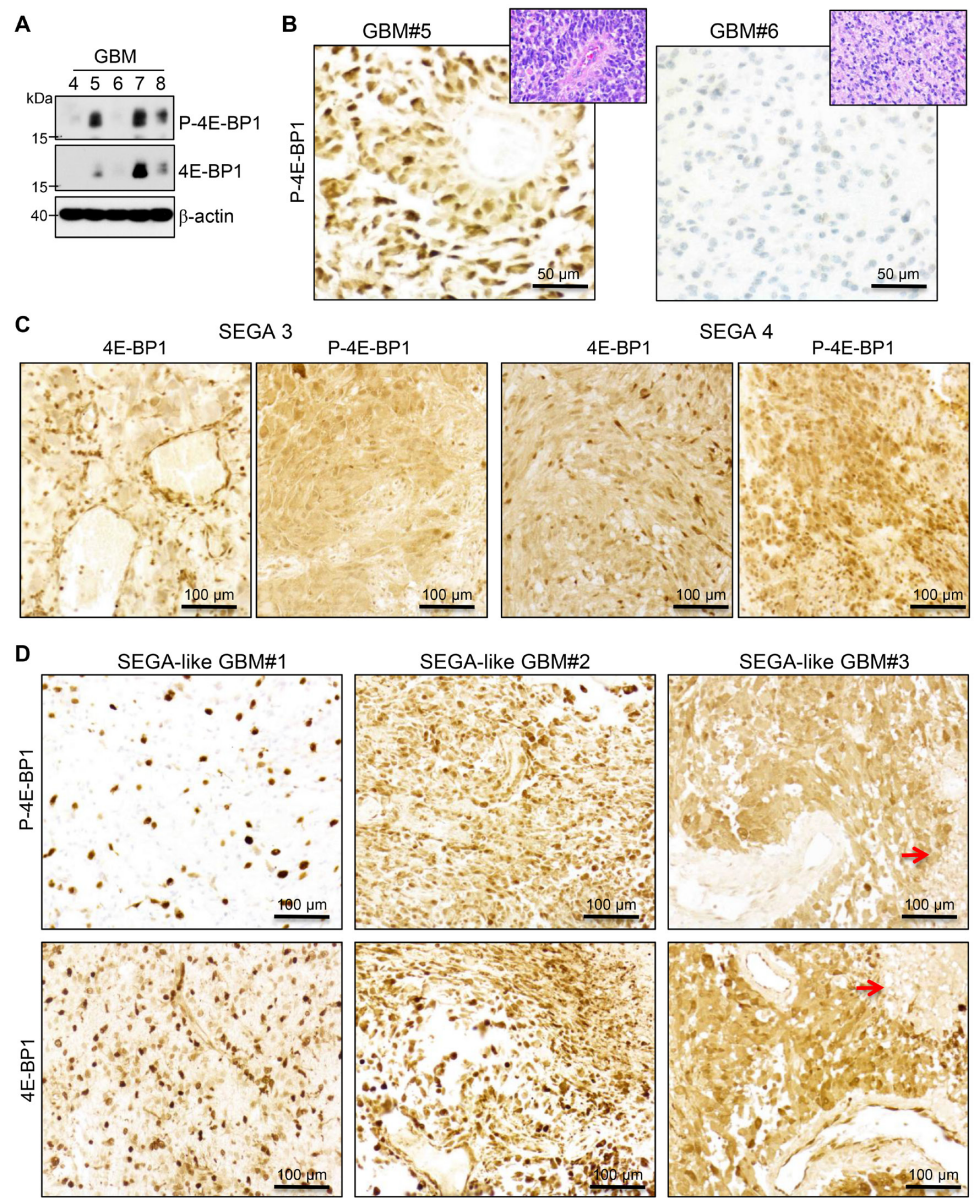
MTOR activation in SEGA and SEGA-like glioblastoma. (**A**) WB analysis with phospho (P)-4E-BP1, 4E-BP1 and beta-actin antibodies of total lysates (30μg) from 5 glioblastoma (GBM) lobar cases, showing variable activation of mTOR. (**B**) IHC with the same P-4E-BP1 on cases #5 and #6, used as positive and negative controls, respectively. Matching H&Es are shown in insets. (**C**–**D**). IHC as in (B) in two cases of SEGA (C) labeled as in Supplementary Figure 1 and the three cases of SEGA-like glioblastoma (D). Red arrows indicate tumor necrosis.

To confirm the activation of mTOR in the SEGA-like glioblastomas, we analyzed by IHC the phosphorylation of 4E-BP1, which is a direct substrate of mTOR kinase [23]. In parallel, we also examined the total protein levels of 4E-BP1, following the recommendations of a comprehensive study proposing guidelines for mTOR activation assessment in glioblastoma [24]. The antibodies used were first tested in WB on five adult glioblastomas without alterations of the MAPK/TSC/mTOR pathway by NGS, and were confirmed to recognize a specific band at the expected size for both phosphorylated and total 4E-BP1 (Figure 4A). The levels of phospho-4E-BP1 and 4E-BP1 were variable in the glioblastoma samples. Two samples with high (#5) and low (#6) levels of P-4E-BP1 in WB were used as positive and negative controls for further IHC testing, respectively (Figure 4B), and showed concordant cytoplasmic P-4E-BP1 expression levels. A minimal nonspecific nuclear stain was noted in the negative control.

The SEGA specimens from patients with TSC syndrome (shown in Supplementary Figure 1) were further tested as positive controls and showed strong 4E-BP1 and phospho-4E-BP1 expression (Figure 4C), consistent with expected mTOR kinase activation. The analysis of the three SEGA-like glioblastomas revealed for cases #2 and #3 a similar pattern of phospho-4E-BP1 and 4E-BP1 expression as in SEGAs (Figure 4D), indicating diffuse strong activation of mTOR in these tumors. In contrast, case #1 showed strong expression of phospho-4E-BP1 only in a subset of neoplastic cells, correlating with the presence of a subclonal population of neoplastic cells with *TSC1* mutation and epithelioid morphology.

## DISCUSSION

Glioblastoma is a heterogeneous pathologic entity and efforts are ongoing to subcategorize it based on morphology and mutational profile, in order to offer a therapy-oriented diagnosis. Of the three recognized variants, the epithelioid is the one recently introduced in the 2016 WHO Classification of CNS Tumors [1]. Mutations in B-Raf, notably the V600E mutation, have been found in 50% of the epithelioid glioblastoma cases [2]. We report here that a subset of periventricular glioblastoma also exhibits an epithelioid SEGA-like appearance, and harbors mutations mapping to the MAPK/TSC/mTOR pathway, in addition to other pathogenic alterations detected in high-grade neoplasms. Moreover, we show that the epithelioid morphology is related to mTOR activation by these mutations.

**Figure 5:**
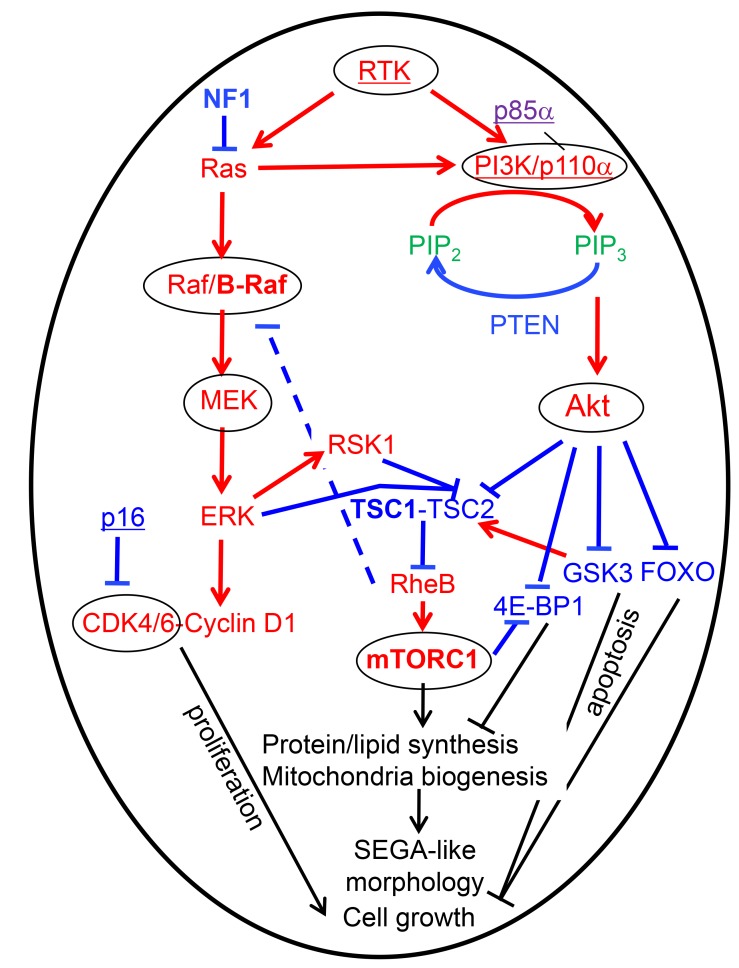
Therapy-oriented mTORC1 signaling model in epithelioid/SEGA-like glioblastoma has been compiled from various sources [3, 9, 25, 46]. Blue labeling, tumor suppressors and inhibitory signaling; punctate line, negative feedback; red labeling, oncogenic proteins and activating signaling; purple, p85α (*PIK3R1*) with dual function; green, signaling lipids; bold letters, the proteins mutated in SEGA-like periventricular glioblastoma (NF1, TSC1 and mTOR from this study, and B-Raf from [6, 34]); underlined, some other mutated proteins contributing to the high grade phenotype; circled, the oncogenic proteins with FDA-approved drug inhibitors; black labeling, cellular effects of the pathways. Note the marked anabolic and biogenetic effects of the mTOR pathway that most likely induce the epithelioid/SEGA-like appearance of the cells in these tumors.

The most compelling evidence that mutations activating the mTOR pathway correlate with the epithelioid/SEGA-like morphology was revealed by the integrated analysis of the 1st patient’s tumor, in which two distinct temporal, morphological and genetic surgical samples were available for analysis. The epithelioid morphology required the presence of a pathogenic *TSC1* mutation, and, in contrast, in the absence of *TSC1* mutation, a fibrillary/myxoid neoplastic morphology was seen. The most interesting aspect was that the *TSC1* mutation co-existed with an already mutated background with driver mutations in *EGFR* and *PIK3CA,* which, from the literature, are also expected to lead to mTORC1 activation [25] (Figure 5). However, in testing for direct phosphorylation of the mTOR target 4E-BP1, only a subset of neoplastic cells showed mTOR activation, correlating with the subclonal presence of the *TSC1* mutation. The fact that the activation of mTORC1 by the PI3K/PTEN/Akt pathway alone does not correlate with epithelioid morphology is supported by the astrocytic/fibrillary appearance of the overwhelming majority of glioblastoma cases, which belong to the so-called “classic” variant and exhibit PI3K/PTEN/Akt pathway activation, mainly via inactivation of PTEN tumor suppressor [26–30]. It is important, however, to recognize that the PI3K/PTEN/Akt pathway is not a linear pathway and it branches into different growth promoting pathways, of which only one is mTOR [25]. Of note also is that, being non-linear, simultaneous additive mutations along the PI3K/PTEN/Akt pathway have also been described, and this suggests non-redundant selective activation of different downstream branches [25]. In particular, the co-occurrence of *TSC1* and *PIK3CA* or *PTEN* mutations has been reported in bladder cancer [31]. For therapy purposes, the conclusion of these studies, as well as that from our previous study modeling glioblastoma in a mouse model, is that in gliomas with multiple hits, combination therapy targeting different levels might be effective [25, 31, 32] (Figure 5). Another take-out message is that direct testing for mTOR activation, which in our case was carried out by combined 4E-BP1 IHC or WB, should be performed for personalized enrollment of patients in mTOR inhibitor trials, as it may reveal tumor populations or subpopulations that do not harbor mTOR activation. Random patient selection without prior testing for exclusion of patients with tumors without mTOR activation may be responsible for the poor response found in the recent RTOG 0193 trial for glioblastoma patients treated with an mTOR inhibitor [33].

A striking epithelioid SEGA-like morphology and strong mTOR activation were diffusely noted in our 3rd patient’s tumor. The only morphologically and mTOR-activation relevant mutation found in the tumor was in *MTOR*, and it was also consistent with a driver mutation. In addition, considering its allele frequency, this variant may be germline, rather than somatic, although we did not have the patient’s normal sample for definitive confirmation of this assumption. Interestingly non-recurrent mTOR mutations scattered over the C-terminus have been reported in renal cell carcinoma [19]. In an elegant study, Grabiner et al. showed that these mutations, including neighboring mutations of Q2499R, are pathogenic by reducing the binding to mTOR to its inhibitor, Deptor, and by increasing the resistance of cells expressing these mutants to nutrient deprivation [18]. Interestingly, these mutations did not confer resistance to mTOR inhibitors [18], strongly advocating that mTOR inhibition might be beneficial in such cases (Figure 5).

Although we did not encounter periventricular glioblastoma cases with B-Raf V600E mutations and epithelioid morphology in this series, two recent case reports illustrated the presence of periventricular tumors with similar morphologic characteristics to the ones in our series and B-Raf V600E mutations [6, 34]. B-Raf is the effector of Ras, the most upstream activator of the MAPK pathways downstream from growth factor receptors, whose inhibitor, NF1, is a tumor suppressor that is also inactivated in a large variety of cancers [35] (Figure 5). The tumor from our 2^nd^ patient had epithelioid/gemistocytic morphology and two different truncating mutations in the *NF1* tumor suppressor with LOH. Although *NF1* mutations may occur in glioblastoma in conjunction with other mutations, a fibrillary/piloid, not an epithelioid morphology was associated with them [36]. In our additional eight *NF1*-mutated cases, *NF1* pathogenic mutations were also associated with spindle cell morphology in most of the cases, although epithelioid, giant cell or gemistocytic morphologies were also seen in isolated cases. Syndromic *NF1* germline mutations, similar to syndromic *TSC1/2* mutations, are associated with low-grade gliomas that nevertheless differ in morphological appearance, with NF1 usually associated with pilocytic astrocytoma, and TSC with SEGA [1]. Surprisingly, the rare glioblastomas arising in patients with NF1 syndrome exhibit an epithelioid morphology, indistinguishable from epithelioid glioblastoma [37, 38]. The epithelioid morphology may result from inactivation of both *NF1* alleles in the presence of an intact downstream pathway resulting in strong activation of mTOR similar to SEGA, where a germline TSC mutation is accompanied by LOH [7]. This mTOR activation would, in turn, impart a plump, morphology to the cells by upregulating a strong biogenetic program (Figure 5). It is not entirely clear why the morphology is variable in tumors with *NF1* mutations but several factors may contribute, including the presence of other genetic alterations, the sequence in which the mutations occur in the tumor and possibly, the cell of origin. For example, we may speculate that the periventricular glioneuronal progenitor cell population that gives rise to SEGA may also be susceptible to the development of a SEGA-like epithelioid glioblastoma if an initiating mTOR pathway mutation is followed by genetic alterations found in high grade gliomas. In other locations where different astrocytic populations reside, *NF1* mutations or even the very rare *TSC* or *MTOR* alterations, may or may not result in an epithelioid morphology, depending on the initiating event and constellation of additional mutations. In-depth studies with concomitant radiologic, genetic, morphological and pathway activation mapping are warranted to further stratify clinico-pathologic subsets of glioblastoma.

In this study, we report a new phenotypic subset of high-grade glioma in which epithelioid SEGA-like morphology is paired with periventricular anatomic location and mutations in genes that activate the MAPK/TSC/mTOR pathway, in addition to other concurrent mutations commonly seen in high-grade neoplasms. These findings highlight three new genes as genotypic-phenotypic associations in epithelioid glioblastoma, in addition to the known alteration of B-Raf V600E. The prognosis was poor, with rapid demise in two patients with subtotal resection, and a course similar to secondary glioblastoma in the remaining patient who has undergone gross total resection. Our findings also reveal drug-actionable mutations [3], and advocate for therapeutic targeting of the MAPK/TSC/mTOR pathway for high-grade epithelioid gliomas that may otherwise behave aggressively when treated only with conventional therapy.

## MATERIALS AND METHODS

### Patients and specimens

Specimens from brain tumors resected in 2009, 2016 and 2017 from three adult white/Caucasian patients aged 46, 60 and 65 years old at the time of surgery were processed in accordance to the LSU-HSC/Shreveport regulations. The selection of these specimens for this study was based on their epithelioid morphology, assessed at the time of the diagnosis by Dr. Georgescu. Magnetic resonance imaging (MRI) studies were performed for all the patients: the reports were available for all the patients, the images were available for the two most recent patients. Formalin-fixed paraffin-embedded (FFPE) sections were stained with hematoxylin-eosin (H&E) and images were acquired at various magnifications with a Nikon Eclipse Ci microscope equipped with a Nikon Digital Sight DS-Fi2 camera (Nikon Instruments Inc., Melville, NY, USA), as previously described [39]. The two most recent cases, as well as additional 50 adult glioblastoma cases from a 2016-2019 prospective cohort from the same institution used for comparison, were diagnosed following the guidelines of the 2016 WHO Classification of Tumors of the CNS [1].

### Immunohistochemistry (IHC) and western blot (WB)

IHC was performed with clinically validated antibodies as previously described [39]. The primary antibodies used were: TTF-1 (clone 8G7G3-1), histone H3 K27M (Millipore Sigma, Burlington, MA), IDH1-R132H (DIA-H09, Dianova, Hamburg, Germany), p53 (DO-7), Ki-67 antibody (30-9) (Roche/Ventana Medical Systems Inc., Tucson, AZ, USA), and GFAP (EP672Y) (Ventana/Cell Marque, Rocklin, CA, USA). The automated Ki-67 proliferation index was performed as previously described, by using the Nikon NIS Elements 4.51.00 program set up with an object count algorithm for recognition of the differentially labeled nuclei [40]. Immunohistochemistry with antibodies for phospho-Thr37/46-4E-BP1 (236B4) and total 4E-BP1 (53H11) (Cell Signaling Technology, Danvers, MA, USA) at 1:1000 and 1:2000 dilution, respectively, was manually performed as described [41]. Tumor tissue lysates from fresh frozen glioblastoma and WB were performed as previously described [42]. The primary antibodies phospho-4E-BP1 and total 4E-BP1 were the same as used for IHC. Beta-actin (AC15; Sigma-Aldrich, St Louis, MO, USA) was used as loading control.

### Nucleic acid preparation for next generation sequencing (NGS)

Nucleic acids were extracted from FFPE samples. FFPE tissue scrolls (50 μm-thick) were deparaffinized and genomic DNA was extracted by using AllPrep DNA/RNA FFPE Kit (Qiagen, Hilden, Germany), following the manufacturer’s instructions. Extracted DNA was quantified with a Qubit fluorimeter (Invitrogen, Carlsbad, CA, USA) by using Qubit BR dsDNA Assay Kit. The fragmentation of the extracted nucleic acids was determined by using the Illumina TruSeq^®^ FFPE DNA Library Prep QC Kit qPCR-based assay (Illumina, San Diego, CA, USA) and the resulting ΔΔCq DNA integrity score and precise quantity of amplifiable DNA were used for DNA input recommendations. The samples were further sheared on a Covaris M220 (Covaris, Woburn, MA, USA) in order to obtain 150-200 bp DNA fragments. The DNA integrity and fragment size were further determined on a Bioanalyzer 2100 (Agilent, Santa Clara, CA, USA).

### NGS library design and preparation

A 295-gene library containing genes with recurrent mutations in adult and pediatric, primary and metastatic brain cancer was designed (Supplementary Table 1). The DNA library was generated with 97–100% gene coverage by Agilent, based on hybrid-capture/target-enrichment SureSelect XT HS technology that yields high QC values and good on-target rates. The entire workflow for DNA library preparation followed the manufacturer’s protocols. The concentration of the samples with captured DNA libraries was determined by using a qPCR-based assay (NEBNext^®^ Library Quant Kit, New England Biolabs, Ipswich, MA, USA). A final concentration of 4 nM was obtained for each library sample, and 5 μl aliquots were pooled, mixed with a 150-cycle High output cartridge (Illumina) and subjected to sequencing on a NextSeq 550 instrument (Illumina). Paired-end sequencing (read length of 2 × 75 bp) was used to improve the sensitivity of duplicate detection.

### NGS analysis, validation and terminology

Raw FASTQ files were analyzed initially with the SureCall program (Agilent) and subsequently exported and analyzed in Microsoft-Excel. Variants were identified through aligning the patient’s DNA sequence to the human genome reference sequence version hg19 (GRCh37). Cut-off minimal values per specimen for confident interpretation of the NGS data were set at 100 for filtered read depth, and 0.1 for allele frequency. Ten representative cases, including the one from the 3rd patient in this study, were cross-sequenced at Tempus Labs (Chicago, IL, USA), and yielded identical results for the common genes present in the two libraries. Additional 50 glioblastoma cases from the 2016–2019 prospective cohort were analyzed by NGS performed at Tempus Labs. The identified mutations per patient are given in Supplementary Table 3. To exclude common population single nucleotide polymorphism (SNP) variants, an ad-hoc database was generated from the initial 112 brain tumor and normal samples NGS results. For clinical significance, the specific SNPs were compared with public databases of known SNPs, including ClinVar (National Institutes of Health) and Catalogue of Somatic Mutations in Cancer (COSMIC), among others. For interpreting the somatic versus germline origin of the mutations, the tumor cell versus total cell content ratios expressed in percentage were estimated at 70%, 80%, 50% and 80% (80% also confirmed at Tempus from the same tissue block) for specimens in the order from Table 2, respectively. The mutations were considered somatic heterozygous if the allelic frequency was roughly equal to half of the tumor cell content ratio, and germline SNPs were considered when the allele frequency was around 0.5. The mutations present in a subpopulation of tumor cells were considered subclonal. Graphs were plotted by using the GraphPad Prism program (GraphPad Software, La Jolla, CA, USA).

### SNP-microarray

Microarray-based chromosome analysis of copy number was performed using the IScan^®^ System with the CytoSNP-850K v1.1 BeadChip (Illumina) and analyzed using GenomeStudio (Illumina) and Nexus, version 9.0 (BioDiscovery, Inc, El Segundo, CA, USA) software. Copy number changes, i.e., clonal changes in less than 100% of cells including deletion, duplication, loss of heterozygosity and ploidy, were determined by using the signal intensity determined by the log^2^ ratio along with the B-allele frequency, as described [43].

### Three dimensional (3D) modeling

The various residues mutated in human cancer were mapped and aligned in the 3D structure of the human mTOR kinase domain (Protein Data Base accession number: 4jsn). The models of wild-type and mutated residues were generated by using PyMol Molecular Graphics System (Version 2.2.3, Schrodinger, LLC), as previously described [44, 45].

## SUPPLEMENTARY MATERIALS



## Abbreviations

CNS: central nervous system
DIPG: diffuse intrinsic pontine glioma
EGFR: epidermal growth factor receptor
H&E: hematoxylin eosin
NF1: neurofibromatosis 1gene or product
MAPK: mitogen activated protein kinase
NGS: next generation sequncing
mTOR/*MTOR*: mammalian target of rapamycin
mTORC1: mTOR complex 1
PI3K/*PIK3CA*: phosphatidylinositol 3-OH kinase catalytic subunit alpha
RTK: receptor tyrosine kinase
SNP: single nucleotide polymorphism
TSC: tuberous sclerosis complex
WB: western blot
WHO: World Health Organization
