# Supplementary material for: Mutations of the MAPK/TSC/mTOR pathway characterize periventricular glioblastoma with epithelioid SEGA-like morphology–morphological and therapeutic implications
Source: Oncotarget. 2019 Jun 18;10(40):4038–52. doi: 10.18632/oncotarget.27005 (PMC6592288; doi:10.18632/oncotarget.27005)
Supplement: Supplementary file 1 [file oncotarget-10-4038-s001.pdf]

Mutations of the MAPK/TSC/mTOR pathway characterize periventricular glioblastoma with epithelioid SEGA-like morphology–morphological and therapeutic implications

SUPPLEMENTARY MATERIALS

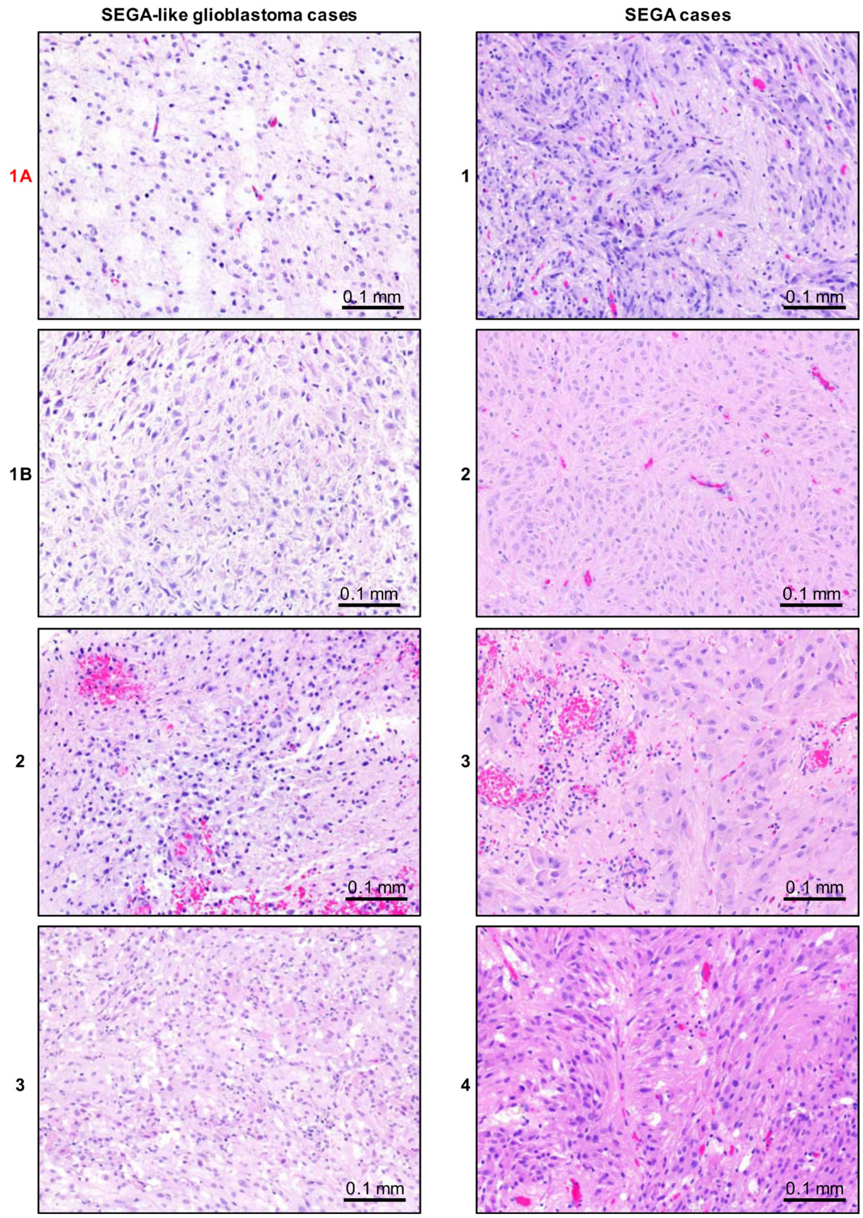

Supplementary Figure 1: Histological comparison between the three SEGA-like glioblastoma cases and 4 SEGA cases. H&E shows similar morphology between SEGA and SEGA-like glioblastoma cases #2 and #3. For the patient #1 with two resections, the SEGA-like epithelioid morphology is confined to the tumor #1B.

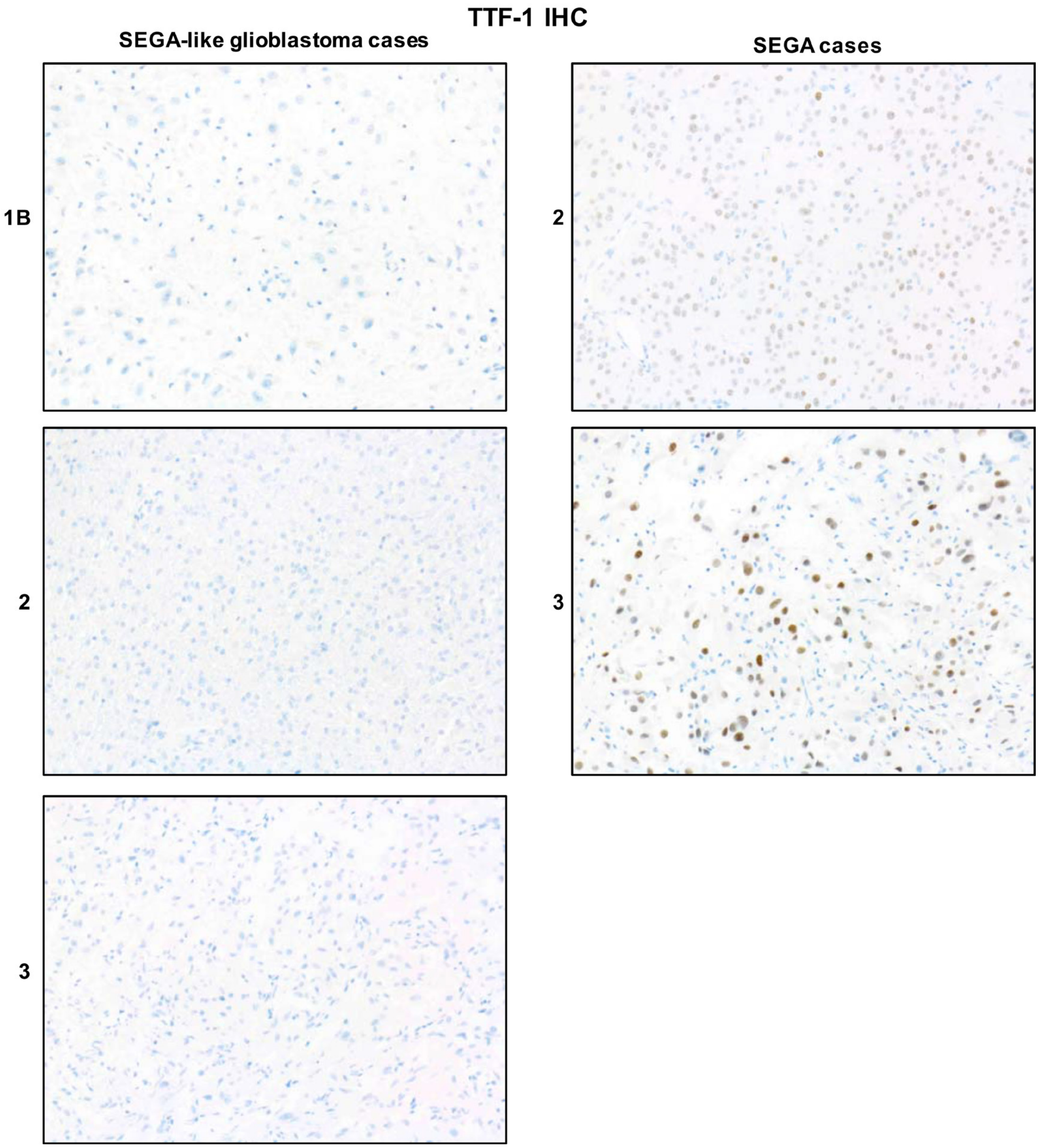

Supplementary Figure 2: SEGA-like glioblastoma cases are negative for TTF-1 expression. IHC with TTF-1 antibody shows variable nuclear expression in SEGA cases and lack of labeling in SEGA-like glioblastoma cases.

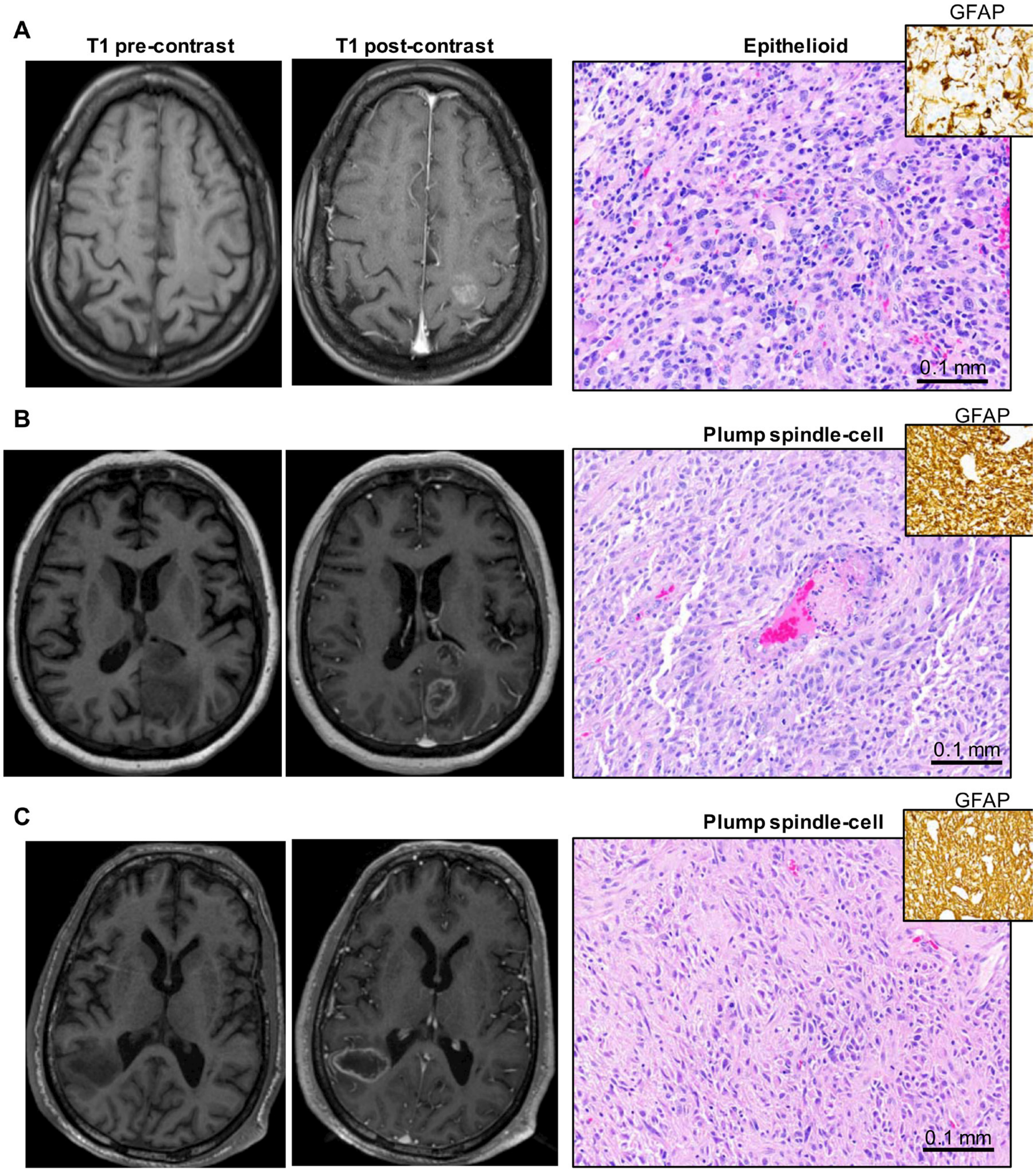

Supplementary Figure 3: MRI and histological appearance of 3 additional glioblastoma cases with NF1 pathogenic mutations accompanied by LOH. The insets show GFAP IHC: mainly negative in the epithelioid tumor from (**A**) and positive in the tumors with plump spindle cell morphology from (**B**) and (**C**). Additional MAPK/TSC/mTOR pathway mutations are present in the tumors from (B) (*MTOR* splice variant) and (C) (*TSC2* indel).

Supplementary Table 1: Next generation sequencing gene panel

ACTB	ATR	CDK4	DDIT3	FAT1	GMPS	IGF2R	LRP1B	MTOR	NTRK1	POLE	RASA1	SMARCA4	TCF12
ACVR1	ATRX	CDK6	DDX3X	FAT2	GNA11	JAK1	LZTR1	Mutyh	NTRK2	POLR2A	RB1	SMARCA5	TEAD1
AFF3	AXL	CDKN2A	DICER1	FBXW7	GNAQ	JAK2	MACF1	MYB	NTRK3	POT1	RDX	SMARCB1	TENM1
CCNB3	BAP1	CDKN2B	DUX4	FEV	GNAS	JAK3	MAGED2	MYBL1	NUTM1	POU2AF1	RELA	SMARCE1	TERT
CDH11	BCHE	CHD7	EDIL3	FGFR1	H3F3A	JAZF1	MAGEH1	MYC	Olig2	POU5F1	RET	SMO	TET2
AIP	BCOR	CHEK2	EGFR	FGFR2	HDAC2	JMJD1C	MAML2	MYCN	PALB2	PPM1D	RIMS2	SNCAIP	TFE3
AKT1	BCORL1	CHTOP	ELAC2	FGFR3	HDAC9	JPX	MAP2K1	NAB2	PAX3	PRKAR1A	RIPK4	SOX4	TFG
AKT2	BEND2	CIC	ERBB2	FGFR4	HEY1	KDM5A	MAX	NAV1	PAX7	PRKCA	RNF130	SRF	TP53
AKT3	BRAF	COL6A3	ERBB3	FLI1	HIST1H3B	KDM5C	MDM2	NCOA1	PDGFB	PRNP	ROS1	SS18	TRAF7
ALK	BRCA1	CREB1	ERBB4	FOSB	HIST1H3C	KDM6A	MDM4	NCOA2	PDGFRA	PSEN1	RPS6KB2	SS18L1	TSC1
APC	BRCA2	CREB3L1	ERG	FOXO1	HMGA1	KDR	MEK1	NF1	PDGFRB	PSEN2	SDHA	SSX1	TSC2
APP	BRD4	CREB3L2	ERRFI1	FOXO4	HMGA2	KIAA1549	MEN1	NF2	PHF1	PTCH1	SDHB	SSX2	TTYH1
AR	BRIP1	CREBBP	ESR1	FOXP1	HOXB13	KIT	MET	NFKBIA	PHLPP1	PTEN	SDHC	SSX4	UGT8
ARHGEF2	C11orf95	CRISPLD1	ETS1	FOXR1	HRAS	KLF17	Mir19MC	NOTCH1	PHLPP2	PTPN11	SDHD	STAG2	USP6
ARID1A	CAMTA1	CSF1	ETV1	FOXR2	ID2	KLF4	MKL1	NOTCH3	PIK3CA	PTPRD	SERPINE1	STAT6	VGLL3
ARRDC4	CCND1	CSF1R	ETV4	FUBP1	IDH1	KMT2B	MLH1	NOTCH4	PIK3R1	PTPRR	SETD2	STK11	VHL
ASPSCR1	CCND2	CTNNB1	ETV5	FUS	IDH2	KMT2C	MMP16	NR3C2	PKIA	PVT1	SLC44A1	SUFU	Vil2 (EZR)
ASXL1	CCND3	CTNNA2	ETV6	GABRG1	IDH3A	KMT2D	MN1	NR4A3	PKM2	RAD51B	SLC9A3R1	SUZ12	WT1
ATF1	CD74	CXXC5	EWSR1	GFAP	IDH3B	KRAS	MSH2	NRAS	PLAG1	RAD51C	SLC9A3R2	TACC1	YAP1
ATG7	CDH1	D2HGDH	EZH2	GLI1	IDH3G	L2HGDH	MSH6	NRG1	PLK2	RAD51D	SLMAP	TACC3	YWHAE
ATM	CDK12	DAXX	FAM131B	GLI2	IGF1R	LDB1	MSN	NSD1	PMS2	RAF1	SMAD4	TAF15	ZPF36

Supplementary Table 2: SNP-microarray data

Patient 1:

Chr	Cyto-bands	Start	Stop	CN	Comments
5	p15.33-q35.3	1	180915260	4	50% high copy gain 5
7	p22.3-p11.1	1	58011874	5	50% high copy gain 7p (including EGFR, PMS2)
7	q11.1-q36.3	61068063	159138663	4	50% high copy gain 7q
9	p24.3-q34.3	1	141213431	1	50% deletion 9 (including TSC1)
14	q11.2-q24.1	19280733	69055799	1	50% deletion 14q
20	p13-q13.33	1	63025520	3	50% gain 20
21	q11.2-q22.3	14613203	48129895	3	50% gain 21
22	q11.1-q13.33	16114244	51304566	1	50% deletion 22

Patient 2:

Chr	Cyto-bands	Start	Stop	CN	Comments
1	p36.33-p36.21	1	13279888	1	20% deletion 1p
4	q22.3	95991256	96437578	1	40% deletion 4q
6	p25.3-q27	n/a	n/a	1	40% deletion 6
9	p22.1	18979004	19392578	1	40% deletion 9p
9	p22.1-p22.3	19830000	19915526	1	40% deletion 9p
9	p21.3	21329044	21508325	1	40% deletion 9p
9	p21.3	21806130	22280635	0	40% homozygous deletion 9p incl. CDKN2A/B
9	p21.3	22280636	22607394	1	40% deletion 9p
9	p21.3	22821568	23287401	1	40% deletion 9p
9	p21.3	25226340	25332933	1	40% deletion 9p
9	p21.2	25662011	26775507	1	40% deletion 9p
9	p21.2	27038294	27164110	1	40% deletion 9p
10	p15.3-q26.3	n/a	n/a	1	40% deletion 10
12	q21.2	76124158	77799670	1	40% deletion 12q
13	q11-q34	n/a	n/a	1	40% deletion 13
14	q11.1-q32.33	n/a	n/a	1	40% deletion 14
18	p11.32-p11.21	1	12917179	1	40% deletion 18p
18	q22.1-q23	63679544 7	8048395	1	40% deletion 18q
21	q21.1-q21.2	20142202	24691022	1	40% deletion 21q
21	q21.2	25016350	25550100	1	40% deletion 21q
21	q25.3-q26.11	25913018	26166103	1	40% deletion 21q
22	q12.2-q12.3	31336780	32735768	1	40% deletion 22q
22	q12.3	33010362	33320271	1	40% deletion 22q
22	q12.3-q13.2	35246521	44107175	1	40% deletion 22q

Patient 3:

Chr	Cyto-bands	Start	Stop	CN	Comments
1	q22-q32.1	156018183	205921859	2	20% LOH 1q
1	q42.13-q43	228520973	241034693	2	20% LOH 1q
6	p25.3-q27	1	171115067	1	20% deletion 6
7	p22.3-q36.3	1	159138663	3	20% gain 7
9	p24.3-q34.3	1	141213431	1	20% deletion 9 (including TSC1)
10	p15.3-q26.3	1	135534747	1	20% deletion 10 (including PTEN)
13	q11-q34	19147562	115169878	1	20% deletion 13 (including RB1)
14	q11.2-q32.33	19280733	107349540	1	20% deletion 14
16	p13.3-q24.3	1	90354753	1	20% deletion 16 (including TSC2)
22	q11.1-q13.33	16197021	51304566	1	20% deletion 22

Start and stop positions are given relative to [GRCh37]. **Abbreviations:** CN, copy number; LOH, loss of heterozygosity.

Supplementary Table 3: Mutations in SEGA-like glioblastoma

Patient	Gene	Nucleotide	Amino acid	Effect	Allele Frequency	NM
#1	PIK3CA	c353G>A	G118D	Misssense	0.387	NM_006218.ex.3
	PMS2	c.2380C>T	P794S	Misssense	0.216	NM_000535.ex.14
	EGFR	c.866C>T	A289V	Misssense	0.364	NM_005228.ex.7
	TSC1	c.395G>A	G132D	Misssense	0.202	NM_000368.ex.6
#2	NF1	c.5734.G>T	G1912*	Stop gain	0.235	NM_000267.ex.38
	NF1	c.6388insT	I2130 + 11fs	frame shift	0.21	NM_000267.ex.41
	PIK3R1	c.1374-3delAAA	EK458E	In frame deletion	0.209	NM_181524.ex.5
#3	MTOR	c.7496A>G	Q2499R	Misssense	0.49	NM_004958.ex.56
	RB1	c.763C>T	R255*	Stop gain	0.079	NM_000321
	TSC1	c.2356C>T	R786*	Stop gain	0.033	NM_000368
	VHL	c.172C>T	R58W	Missense	0.057	NM_000551
	ETS2	c.421-4delT		Splice	0.182	NM_001256295
	SGK1	c.514-6_514-5delTT		Splice	0.136	NM_001143676
	AXL	c.1446-3delC		Splice	0.053	NM_021913
	MIB1	c.1372-11_1372-4delAAAAAATGinsTTTTGTTT		Splice	0.051	NM_020774
